# Admissions, mortality and financial burden associated with acute hospitalisations for sepsis between 2006 and 2018: A national population-level study

**DOI:** 10.1177/17511437251326774

**Published:** 2025-03-16

**Authors:** Tamas Szakmany, Rowena Bailey, Rowena Griffiths, Richard Pugh, Joe Hollinghurst, Ashley Akbari, Ronan A Lyons

**Affiliations:** 1 Department of Anaesthesia, Intensive Care and Pain Medicine, Division of Population Medicine, Cardiff University, Cardiff, UK; 2 Critical Care Directorate, Grange University Hospital, Aneurin Bevan University Health Board, Cwmbran, UK; 3 Population Data Science, Swansea University Medical School, Faculty of Medicine, Health and Life Science, Swansea University, Swansea, UK; 4 Department of Anaesthetics, Glan Clwyd Hospital, Betsi Cadwaladr University Health Board, Rhyl, UK

**Keywords:** Sepsis, ICD-10 code, mortality, burden, hospitalisation

## Abstract

**Background:**

We assessed the healthcare and economic burden of sepsis in adult hospitalised patients in Wales, UK.

**Methods:**

We analysed hospital admissions to all acute hospitals in Wales via the Secure Anonymised Information Linkage Databank. We included all adult patients, 2006–2018, with an inpatient admission including one or more explicit sepsis codes.

**Results:**

38,564 patients had at least one admission for sepsis between 2006 and 2018. Most persons (86.7%) had just one admission. 3398 patients (8.4%) were admitted to ICU. The number of admissions increased yearly over the study period from 1548 in 2006 to 8708 in 2018. The largest annual increase (141.7% compared to the previous year) occurred in 2017. Admission numbers increased disproportionately amongst patients with high levels of comorbidities, but changes were consistent across all age groups, areas of deprivation and ICU admissions. Estimated inpatient sepsis costs were £340.34 million in total during the study period. The average cost per hospital spell was £7270. Patients readmitted to the hospital for sepsis amassed estimated treatment costs of over £72 million during the study period. Out of the 38,564 persons, 21,275 (55.2%) died within 3 years of their first admission. Inpatient mortality halved from 40.5% to 19.5%, and there was a trend towards reduced mortality at 6 months, 1 and 3 years post hospital discharge.

**Conclusion:**

Sepsis related hospital admissions are increasing over time and still likely to be underreported. Although mortality appears to have fallen, prolonged hospitalisation and readmissions place a significant burden on healthcare system resources and costs.

## Introduction

Sepsis, most recently defined as a dysregulated host response to infection leading to organ dysfunction, is a syndromic illness affecting significant percentages of hospitalised patients in the UK and worldwide.^1–3^ The burden of sepsis continues to grow despite advances in treatments and falling mortality.^1,4^ It is recognised that a significant proportion of the cost and societal implications will fall outside the acute hospitalisation phase.^5^

Much work has been done on understanding predictors of developing sepsis and the mortality associated with the condition, both in the short and longer term.^6^ It is recognised that those who are older, have significant chronic health conditions and need support for normal activities of daily living are at highest risk.^2,6^ Measuring the burden of sepsis is fraught with difficulty, as the current clinical definition uses complex physiological data, which is rarely available outside of the critical care environment.^3,7^ The World Health Organisation (WHO) created the International Classification of Diseases (ICD), a standardised set of codes used worldwide.^8^ The current 10th iteration of ICD codes (ICD-10) has been used in several epidemiological sepsis studies despite the tendency to under-code the sepsis episode in administrative databases.^9–11^

In this study, we aimed to assess the healthcare and economic burden of sepsis in hospitalised patients in Wales, UK, and to understand their relationships with illness severity, chronic health and sociodemographic factors.

## Methods

### Reporting guidelines and conventions

This study has been reported according to the Strengthening the Reporting of Observational Studies in Epidemiology (STROBE) and REporting of studies Conducted using Observational Routinely collected Data (RECORD) guidelines.^12,13^

### Data

All de-identified data were accessed and analysed within the Secure Anonymised Information Linkage (SAIL) Databank (www.saildatabank.com). A detailed description of SAIL and the individual data sources used for this study is provided in the Supplemental Digital Content (SDC).

### Study cohort

Patients over 16 years of age and resident in Wales were included in the cohort if they had a hospital admission with a diagnosis of any of the ICD-10 sepsis codes (see Supplemental Table 1 for complete list of codes) between April 2006 and December 2018, and a known discharge date. Individuals with missing demographic data were excluded.

### Outcomes

The study’s primary outcome was the number of hospital admissions related to sepsis. Multiple admissions for the same individual within the study period were retained. Mortality following admission was derived from the difference between the discharge date and the date of death and categorised in intervals at 7 days, 6 months, 1 and 3 years from discharge. Where the hospital discharge date and date of death were the same, it was inferred that the patient died before discharge. Patients were followed up for 3 years until December 2021 using the Annual District Death Extract database from the Office for National Statistics.

### Variables

The year of admission was derived from the admission date using calendar years. Sepsis coding criteria were revised in April 2017 to emphasise clinical terminology used and were further modified in April 2018 to address the vague terminology associated with local infections.^14^

Hospital admissions were flagged if there was a corresponding stay in Critical Care (CC). A CC stay was determined to be associated if the CC admission date was between the admission and discharge dates or within 7 days of the admission or discharge dates. Severity of sepsis was categorised based on CC admission during the sepsis related hospital admission. We further categorised admissions according to the presence of specific causative organism (sepsis subtype), comorbidity measured by the Charlson comorbidity index (CCI), frailty using the electronic Frailty Index (eFI) and socioeconomic deprivation characterized by the Welsh Index of Multiple Deprivation, 2014 (WMID).^15–17^ Detailed description of the CCI, eFI and WIMD is provided in the SDC.

Length of Stay (LOS) was calculated as the number of days between admission and discharge dates. LOS was imputed as 1 day where the dates were the same. LOS was grouped for admissions with durations of 1–2, 3–4, 5–6 and 7 days or more. Age was calculated in years at the admission date and grouped as less than 65, 65–74, 75–84, and 85 or above.

Inpatient admission treatment cost estimations were calculated according to the National Schedule of Reference Costs.^18^ The average in-hospital bed day cost was £471/day using the sepsis related currency. The total number of bed days was derived using the total LOS for all admissions, and the total inpatient costs were estimated by multiplying the average cost per day by the total number of bed days.

### Statistical analysis

Descriptive statistics of patient characteristics are presented as counts and percentages of persons within each demographic category as at the date of their first admission. Hospital admissions relating to sepsis were analysed as counts and presented as trends over time using 3 months rolling averages. Hospital admissions are also summarised. Mortality statistics are presented as the percentage of admissions within each interval and derived cumulatively such that percentages for shorter time periods are subsets of longer mortality intervals. Mortality percentages are presented by demographic and clinical sub-group category, and trends over time are presented with 3 months rolling averages.

### Ethics

All data used in this study was anonymised and provisioned within the SAIL Databank. The analysis of anonymized linked data was approved by the Information Governance Review Panel of the SAIL Collaboration Review System (Longitudinal analysis of Critical Care Outcomes in Wales, Project No: 0634, June 20, 2017) and is fully compliant with the Declaration of Helsinki (1975) regarding ethical principles for medical research involving human subjects.

## Results

### Sepsis patients

**Figure 1.**
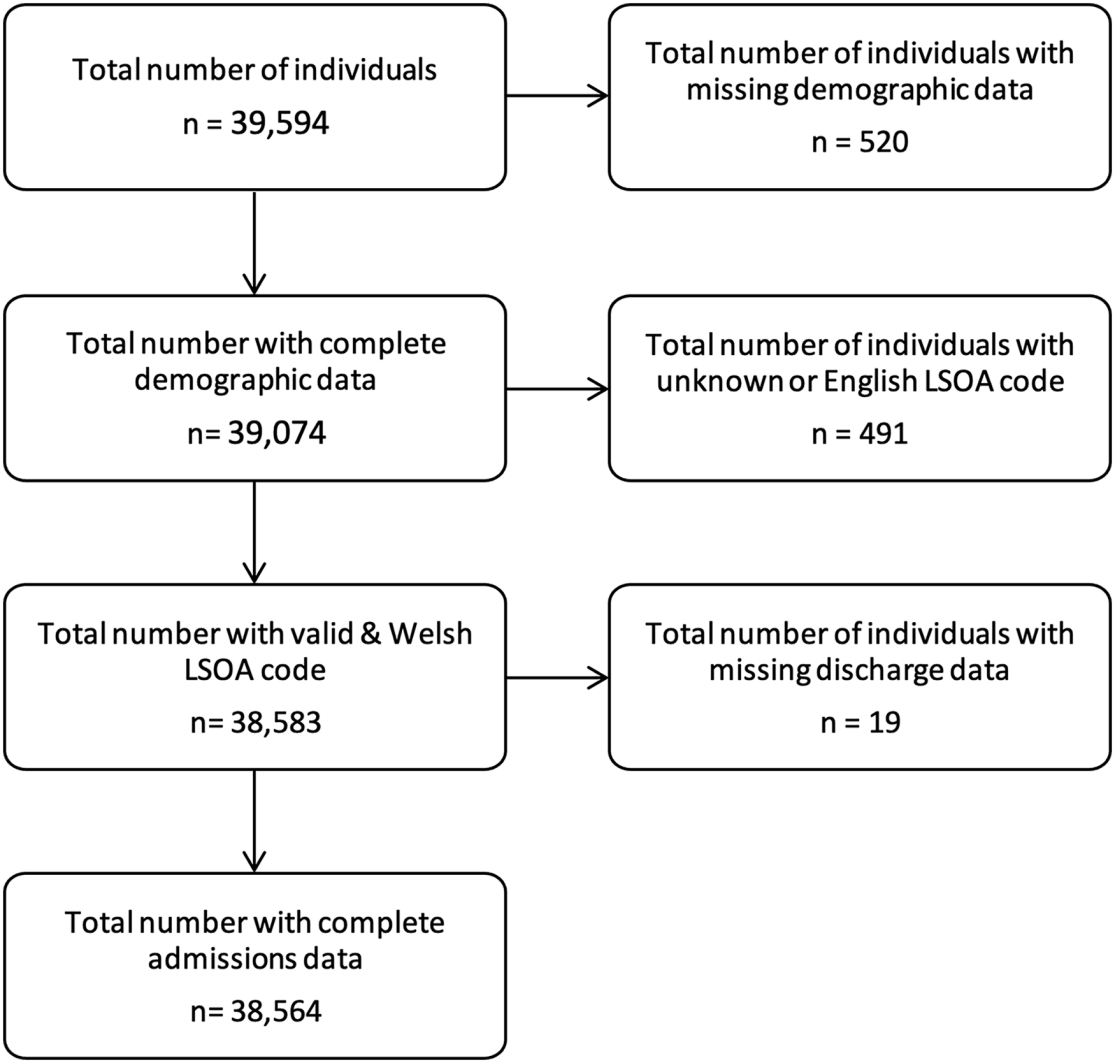
CONSORT diagram for the study. LSOA: local super output area.

The number of persons with at least one admission for sepsis between 2006 and 2018 was 39,594. After excluding records for persons with missing demographic data, persons with unknown or English LSOA codes, and missing hospital discharge information, the final number of persons included in the analysis was 38,564 (Figure 1).

There were more females than males in the cohort (51.2% and 48.8% respectively). Approximately seven out of 10 patients were 65 years old or older. Fewer persons in the cohort lived in the least deprived areas (Table 1). Some 41.2% of the cohort were categorised as ‘fit’ using the eFI score, with missing information for just over 3% of the cohort. 42.5% of the cohort had more than 10 comorbidities, and over one in five patients had no recorded comorbidities (21.6%).

**Table 1.** Distribution of patient characteristics at the date of the first admission.

Patient characteristics	Categories	Number of persons	
		(*n*)	%
Total		38,564	100.0
Sex	Male	18,832	48.8
	Female	19,732	51.2
Age (years)	Under 65	11,302	29.3
	65–74	8502	22.1
	75–84	10,520	27.3
	85 and older	8240	21.4
Age group	Under 65 years	11,302	29.3
	65 years and older	27,262	70.7
WIMD fifths	Most deprived	7831	20.3
	2	8175	21.2
	3	8266	21.4
	4	7665	19.9
	Least deprived	6627	17.2
eFI category	Fit	15,881	41.2
	Mild	10,504	27.2
	Moderate	7558	19.6
	Severe	3387	8.8
	Missing	1234	3.2
Charlson comorbidity index category	Low	5792	15.0
	Medium	8076	20.9
	High	16,371	41.2
	Missing	8325	21.6
Number of admissions	1	33,437	86.7
	2–3	4765	12.4
	4–5	298	0.8
	More than 5	64	0.2

WIMD: Welsh Index of Multiple Deprivation (2014); eFI: electronic frailty index.

### Hospital admissions

The number of hospital admissions was 45,515 over the entire observation period, corresponding to a mean of 1.2 (SD = 0.6) admissions per person. Most persons (86.7%) had just one admission. A small proportion (0.94%) had four or more admissions for sepsis (Table 1). Some 3398 patients (8.4%) were admitted to CC.

**Figure 2.**
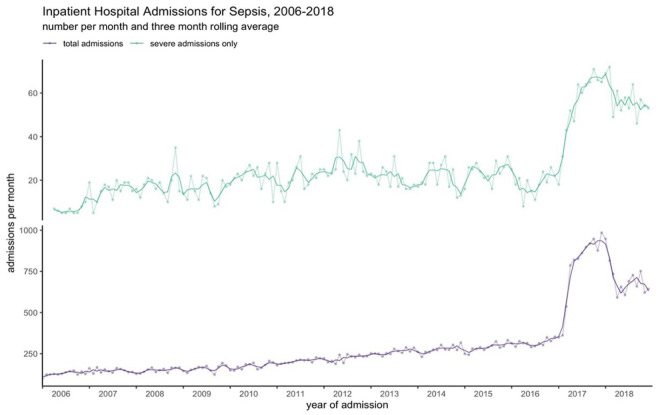
Inpatient hospital admissions with sepsis specific ICD-10 codes in Wales, 2006–2018. Admissions are presented as number per month – dots and three-month rolling average – solid line. Total admissions – purple line; critical care admissions (severe admissions) – green line.

The number of admissions increased yearly over the study period from 1548 in 2006 to 8708 in 2018 (Figure 2 and Supplemental Table 2). The largest annual increase (141.7% compared to the previous year) occurred in 2017, corresponding to clinical coding changes in the same year, decreasing slightly (−7.9%) in 2018 following further revisions to coding standards (Supplemental Table 3).

The changes were consistent across all age groups (Supplemental Figure 1 and Table 4), sex (Supplemental Figure 2 and Table 4), and across areas of deprivation (Supplemental Figure 3 and Table 4). Admission numbers increased disproportionately amongst patients with high levels of comorbidities (Supplemental Figure 4 and Table 4). Increased admissions were mostly seen in patients admitted to the hospital with sepsis without a specific causative organism (Supplemental Figure 5 and Table 4) and in those who were characterised ‘fit’ by the eFI (Supplemental Figure 6 and Table 4). Admissions with a corresponding CC stay also increased, with a 184.6% increase in 2017 (Figure 2 and Supplemental Table 3).

More than half of all admissions lasted more than 7 days (57.7%) (Supplemental Figure 7). Sepsis related inpatient bed days increased yearly until 2017, with a small decrease from 2017 to 2018 (Supplemental Table 5). Sepsis related hospital days increased from 0.66% of all in-hospital days in 2006 to 4.2% in 2017, with a slight decrease to 3.8% in 2018. The estimated inpatient cost of sepsis increased steadily over the study period in line with the number of admissions and total bed days (Supplemental Table 5). Inpatient sepsis costs reached £340.34 million in total during the study period. The average cost per hospital spell was £7270. Patients readmitted to the hospital for sepsis amassed estimated treatment costs of over £72 million during the study period.

The majority of admissions were classified as ‘non-severe sepsis, unspecified’ (70.6%). The detailed breakdown of the number of sepsis related hospital admissions by ICD-10 codes is provided in (Supplemental Table 6). Of note, no patient had ICD-10 admission code R6520 (Severe sepsis without shock) or R6521 (Septic shock) recorded.

### Mortality

**Figure 3.**
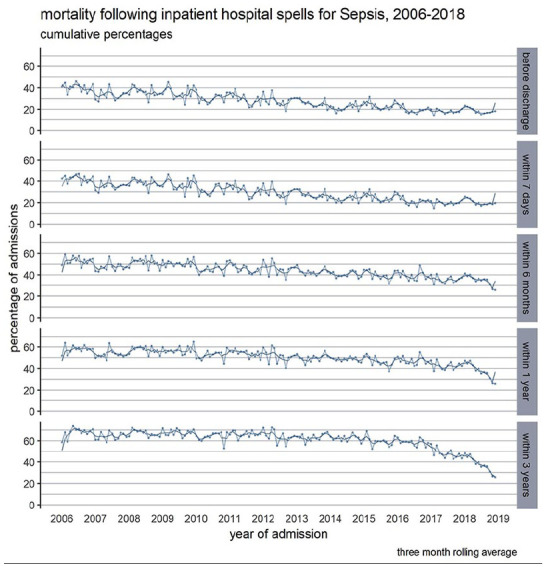
Mortality following in hospital spells with sepsis specific ICD-10 codes in Wales, 2006–2018, before hospital discharge, within 7 days, 6 months, 1 and 3 years following admission.

Out of the 38,564 persons, 21,275 (55.2%) died within 3 years of their first admission. Approximately 1 in 4 (24.7%) of the deceased patients died within 7 days of the index admission (Table 2). Mortality improved over the study period in every timeframe. Inpatient mortality has halved, and there was a trend towards reduced mortality at 6 months, 1 and 3 years (Figure 3). The reduction in mortality was most pronounced in the over 85 years old group and in patients with high levels of comorbidities (Supplemental Figure 8).

**Table 2.** Distribution of clinical characteristics of admissions.

Patient characteristics	Categories	Admissions	
		Number (*n*)	%
Total		45,515	100
Sepsis subtype	Organism specified	13,391	29.4
	Organism unspecified	32,124	70.6
Critical care admission	Yes	3818	8.4
	No	41,697	91.6
Length of stay (days)	1–2	7124	15.7
	3–4	6276	13.8
	5–6	5852	12.9
	7 or more	26,263	57.7
Mortality interval	Died before discharge	11,251	24.7
	Died within 7 days of discharge	852	1.9
	Died within 6 months of discharge	6925	15.2
	Died within 1 year of discharge	2709	6.0
	Died within 3 years of discharge	3924	8.6
	Alive at 3 years after discharge	19,854	43.6

Mortality interval is related to hospital discharge.

The main cause of death was recorded as ICD-10 code A419 (sepsis, unspecified) in 3845 (20.9%) of deaths; J189 (Pneumonia, unspecified) and J180 (Bronchopneumonia, unspecified) were recorded in 1677 (9.1%) and 1460 (7.9%) cases, respectively. Other commonly recorded causes of death were malignancies in 2100 (11.4%), cardiovascular causes in 898 (4.9%) and respiratory causes in 625 (3.4%) patients. The top 10 main causes of death for the cohort are provided in Supplemental Table 7. When analysing the main causes of death, there was a tendency to record A419 in those patients who died early after their admission.

## Discussion

To our knowledge, this is the first ever report on the incidence and outcomes of sepsis in Wales, and currently represents the largest curated dataset available in the UK. We report population-level data in a nation of ~3 million over a 13-year period, where universal health coverage benefits the entire population, and which includes some of the most deprived areas of the UK. Sepsis related admissions increased over the course of the study, with mortality slowly decreasing mostly due to the decrease in early deaths. More than half of the patients spent at least 7 days in the hospital, indicating the burden sepsis places on the NHS, with more than £340 million spent on inpatient care alone over the course of the study.

We aimed to make our results reproducible and comparable to previous studies in this field. To aid this, we followed the methodology of analysing the Medicare dataset previously published by Buchman et al.^5,19^ Hospital admissions with explicit ICD-10 codes for sepsis have grown steadily in line with previously published data in other healthcare environments until 2016.^1^ It has been recognised that using coding and claims data to assess the burden of sepsis only moderately correlates with the use of explicit clinical criteria.^10,20^ In our study, the use of ICD-10 coding to identify sepsis admissions in an routinely-collected, administrative database highlights the profound impact that coding policy changes can have on statistical outcomes. The coding changes introduced in 2017 dictated that if a clinician has used certain terms such as urosepsis, chest sepsis and biliary sepsis, for example, the coders were instructed to use sepsis ICD-10 codes in the primary position of the spell. This change led to a 141.8% increase in recorded sepsis admissions in 2017 compared to the previous year. The estimated incidence of 269 sepsis admissions per 100,000 persons in Wales is still well below the estimated 349 per 100,000 persons in England for the same year.^21^ The exact reason for this discrepancy between England and Wales is not clear, we can only speculate that it may be attributable of different coding practices. Notably, the use of ‘sepsis’ terms instead of codes describing localised infections from April 2017, may have inflated the sepsis admissions recorded in both systems. The 2018 changes attempted to address this and had clearer guidance on addressing the vague terminology of local infections. As a result, sepsis admission numbers reduced slightly (−7.9%) but still were more than double those seen in 2016. Our data suggest that sepsis was significantly underreported before 2017.^9,22^ Sepsis related admissions were more frequent in patients over the age of 65 years, with frailty and with multiple comorbidities. We found a disproportionate increase in admissions for patients with several comorbidities, highlighting the increasing burden of sepsis in this group.

An important observation in our dataset is that less than 9% of the patients with an admission coded with sepsis were admitted to CC. This is at odds with international data from high-income countries, where the admission rate to CC is at least double for such patients.^5,20^ Moreover, a critical care specific dataset from 2009 to 2014 demonstrated that approximately 31% of all CC admissions in England and Wales were due to sepsis.^6^ Our data based on the ICD-10 coding of the hospital admission represents approximately one third of the expected CC case numbers.^23,24^ Furthermore, the absence of admissions with codes for severe sepsis or septic shock suggests a lack of specificity rather than an indication of non-events. The most plausible explanation for this discrepancy is within the process of ICD-10 coding within the Welsh NHS, which clinicians do not conduct. The lack of coding for a condition such as septic shock, which carries high risk of mortality, may have artificially skewed the outcome figures in our dataset. Our data highlights the need for better, physiology-based coding of syndromic diseases such as sepsis and acute respiratory distress syndrome even for administrative purposes.^10^

Sepsis mortality in 2006 was substantially higher than Buchman et al.^5^ reported. We identified a slow but steady decline during the study period, mostly attributable to a greater than 50% reduction in short-term mortality. This stark improvement in short-term survival slowly diminished over the longer-term follow up. A possible explanation is that the biggest improvement in short-term outcomes were seen in elderly patients with high comorbidity. It is plausible that these short-term gains could not be fully translated to better long-term outcomes in this cohort.^25^ This is supported by our own previous data and of others, describing that the preventability of sepsis deaths are more closely linked to the patients’ comorbidity burden and less to the acute infectious episode.^25,26^ Interestingly, in this study we did not observe a significant change in either direction in short term mortality from 2016 despite the large increase in cases identified by the ICD-10 codes. This finding further emphasises the possibility of underreporting of sepsis in the study period.^7,9^ The causes of death in the deceased patients suggest that early mortality was more likely attributed to sepsis or major infectious causes.^26^ The gradual reduction in these deaths over the course of the study suggests better general care processes over the years.^27^ Notably, the National Early Warning Score and its associated triggers for escalation were implemented in 2012 in every acute hospital in Wales.^28^ This year marks the start of a sustained decrease in early deaths in our data, which hasn’t changed significantly in the next period. As similar improvements over time were observed in other countries, we postulate that this reflects general progress in recognition and medical care rather sepsis-specific treatment advances.^25^ Certainly, our yearly point-prevalence studies in sepsis starting in 2015 did not show improved sepsis-specific process in this period.^29^ Importantly, the majority of deaths were coded something other than sepsis. This finding further highlights the imprecision of ICD-10 based diagnostic criteria, as the coded causes of death represented either complications or underlying conditions contributing to sepsis.

More than half of our patients spent over 7 days in-hospital, a stark contrast to the US Medicare dataset, where only one in five patients had similarly long stays.^5^ Although the comorbidity burden was high in our population, and the facilities to discharge patients to intermediate care are limited in Wales, this finding also supports the underreporting of sepsis and the severity of the disease.^9,17^

This study is the first to attempt to estimate the financial burden of sepsis in the secondary care system based on verifiable data in Wales. The steady increase of inpatient costs attributed to sepsis have been shown in other healthcare systems, however the average cost of a sepsis related hospital spell of £7270 ($9480) in the Welsh NHS is lower than previously published US and German data.^5,20^ Given the similarities of longer-term outcomes in these countries, our data provides an important baseline for potential cost-effectiveness comparisons.

Our study has several strengths. We were able to study the whole adult age spectrum in a healthcare system which is universally accessible and free at the point of contact. We have used a previously published methodology to identify sepsis related admissions using ICD-10 codes to make our findings comparable internationally.^30–32^ Our data covers urban, sub-urban and rural areas served with various secondary healthcare settings, making it generalisable. We also acknowledge several limitations in our study. First, the use of routinely-collected electronic health records (EHRs) presents inherent challenges, including potential gaps in available data, variability in data quality, and the possibility of misclassification errors. We were not able to corroborate the admission coding data with detailed physiological information or data on treatment pathways. The lack of clinical data has been shown to significantly impact on the accuracy of diagnosis and subsequent outcomes.^10^ As the switch to digital recording of this data is underway in Wales, we hope to utilise the spread of electronic observations in hospitals to have further insight. Second, there were no patient admissions coded with severe sepsis or septic shock ICD-10 codes. This raises the question about the general quality of the hospital coding.^33^ Third, to describe comorbidities, we used only the Charlson comorbidity index which doesn’t provide granular data on underlying conditions; however, we have shown this index to be the best predictor of mortality in our previous studies in similar settings.^34^ Fourth, we used ICU admission as a surrogate for severity, with limited success. Unfortunately, due to gaps in reporting and data availability issues in the current project, we were unable to investigate this discrepancy further, leading to possible underreporting of case numbers, severity, mortality and costs. Fifth, cost estimates were based on NHS England tariffs and not actual expenditure in the Welsh NHS. Due to data privacy concerns, the direct costs attributed to each hospital spell are currently unavailable in SAIL.

In conclusion, our longitudinal, population-based study found that sepsis related hospital admissions are increasing over time and still likely to be underreported. Although mortality appears to have fallen, this is probably not directly attributable to sepsis-specific care improvements. Prolonged hospitalisation and readmissions place a significant burden on the healthcare system resources and costs. Our data emphasises the needs both to identify and reliably treat sepsis, and to provide appropriate aftercare for patients, which can reduce the long-term burden of the condition. Furthermore, our data can be used in modelling the future burden of sepsis across the adult population.

## Supplemental Material

sj-docx-1-inc-10.1177_17511437251326774 – Supplemental material for Admissions, mortality and financial burden associated with acute hospitalisations for sepsis between 2006 and 2018: A national population-level study Supplemental material, sj-docx-1-inc-10.1177_17511437251326774 for Admissions, mortality and financial burden associated with acute hospitalisations for sepsis between 2006 and 2018: A national population-level study by Tamas Szakmany, Rowena Bailey, Rowena Griffiths, Richard Pugh, Joe Hollinghurst, Ashley Akbari and Ronan A Lyons in Journal of the Intensive Care Society
