# Supplementary material for: Admissions, mortality and financial burden associated with acute hospitalisations for sepsis between 2006 and 2018: A national population-level study
Source: J Intensive Care Soc. 2025 Mar 16;26(3):294–301. doi: 10.1177/17511437251326774 (PMC11912151; doi:10.1177/17511437251326774)
Supplement: sj-docx-1-inc-10.1177_17511437251326774 – Supplemental material for Admissions, mortality and financial burden associated with acute hospitalisations for sepsis between 2006 and 2018: A national population-level study [file sj-docx-1-inc-10.1177_17511437251326774.docx]

Supplementary Digital Content

## Admissions, mortality and financial burden associated with acute hospitalisations for sepsis between 2006 and 2018: A national population-level study

Tamas Szakmany MD, PhD, FCCM^1,2,*^, Rowena Bailey MSc^3,*^, Rowena Griffiths MSc^3^, Richard Pugh MSc, FRCA, FFICM^4^, Joe Hollinghurst PhD^3^, Ashley Akbari MSc^3^, Ronan A Lyons FMedSci^3^

Table of content

Methods page 2-4

eTables 1-7 page 5-15

eFigures 1-8 page 16-23

References page 24-25

Methods

The data used for this study were extracted and analysed from tables held within the Secure Anonymised Information Linkage (SAIL) Databank ([www.saildatabank.com](http://www.saildatabank.com/)).

*SAIL Databank*

The SAIL Databank is a state of the art, remotely accessible, Trusted Research Environment (TRE) accredited under the Digital Economy Act. SAIL is a constantly updated system which covers the current Welsh population of about 3.1 million people with routine electronic health record (EHR) data from primary and secondary health care available. The data are held within the privacy-protecting Secure eResearch Platform (SeRP) based at Swansea University and provides access to linked de-identified data from multiple sources at an individual, household and multiple ecological levels, for the population of Wales (1-3). These anonymised population-scale, individual-level data sources provide large volumes of data and a broad longitudinal view of a condition over long periods of time. The SAIL Databank has a strict set of policies, structures and control practices to protect privacy through a matching, anonymisation and encryption process (1-3). The anonymisation process is conducted by the Digital Health and Care Wales (DHCW), formally known as National Health Service (NHS) Wales Informatics Service (NWIS). It involves splitting any identifiable data from the clinical data and assigning an Anonymised Linkage Field (ALF) to an individual based on several matching criteria. This enables the linkage of cases across multiple data sources acquired and linked into SAIL through matching via their ALF and allows a broad longitudinal view of their conditions and treatments (1-3).

*Datasets*

The Patient Episode Dataset for Wales (PEDW) contain details of all admissions, attendances and appointments. Admissions relating to sepsis were identified using the International Classification of Diseases version 10 (ICD-10) codes (Supplementary Table 1.) recorded in the admissions data.

The Critical Care Data Set (CCDS) is based on the Critical Care Minimum dataset. Since 2007, local health boards are required to submit monthly extracts detailing critical care activity to provide a standardised set of data on patients admitted to a critical care unit in Wales. This dataset was used to identify cases in the sepsis cohort with a critical care spell associated with the sepsis related hospital admission.

The Annual District Death Extract (ADDE) of the Office for National Statistics (ONS) contains mortality information linked to sepsis patients to obtain the date of death and the primary cause of death recorded for registered deaths.

The Welsh Demographic Service Dataset (WDSD) contains demographic details on everyone treated by the NHS. WDSD data were linked to PEDW records to obtain patient demographics. Information included the Week of Birth, sex and Lower-layer Super Output Area (LSOA) of place of residence at the time of admission. LSOAs are a statistical geography units of about 1500 people and were used to map patients to area-level deprivation measures, using the Welsh Index of Multiple Deprivation (WIMD), grouped into fifths (4).

Welsh Longitudinal General Practice (WLGP) dataset contains records from ~80% of General Practices (GP) in Wales regarding patient interactions with primary care services, including GP consultations, prescriptions and referrals. WLGP data is coded using Read v2 codes which capture diagnoses, symptoms and treatments mentioned within patient records.

Derived variables

*electronic Frailty Index (*eFI)

The eFI is based on an internationally established model. It assigns a frailty score to an individual calculated using 36 variables from primary care data, including symptoms, signs, diseases, disabilities and abnormal laboratory values Scores were calculated for individuals on their admission date and categorised as: fit (0 - 0.12), mild (>0.12 – 0.24), moderate (>0.24 – 0.36), and severely frail (>0.36) (5).

*Charlson Comorbidity Index (CCI)*

The modified CCI uses ICD-10 codes and weights detailed in the NHS Summary Hospital-level Mortality Indicator specification to calculate a CCI score for patients at admission (6). Scores were categorised into three groups: low (-1-0), medium (1-10) and high (>10).

eTable 1: ICD-10 codes containing sepsis/septicaemia or SIRS

| ICD-10 code | DESCRIPTION |
| --- | --- |
| A021 | Salmonella sepsis |
| A207 | Septicaemic plague |
| A227 | Anthrax sepsis |
| A267 | Erysipelothrix sepsis |
| A327 | Listerial sepsis |
| A40 | Streptococcal sepsis |
| A400 | Sepsis due to streptococcus, group A |
| A401 | Sepsis due to streptococcus, group B |
| A402 | Sepsis due to streptococcus, group D |
| A403 | Sepsis due to Streptococcus pneumoniae |
| A408 | Other streptococcal sepsis |
| A409 | Streptococcal sepsis, unspecified |
| A41 | Other sepsis |
| A410 | Sepsis due to Staphylococcus aureus |
| A411 | Sepsis due to other specified staphylococcus |
| A412 | Sepsis due to unspecified staphylococcus |
| A413 | Sepsis due to Haemophilus influenzae |
| A414 | Sepsis due to anaerobes |
| A415 | Sepsis due to other Gram-negative organisms |
| A418 | Other specified sepsis |
| A419 | Sepsis, unspecified |
| A427 | Actinomycotic sepsis |
| B377 | Candidal sepsis |
| O85X | Puerperal sepsis |
| R65 | Systemic Inflammatory Response Syndrome [SIRS] |
| R650 | Systemic Inflammatory Response Syndrome of infectious origin without organ failure |
| R651 | Systemic Inflammatory Response Syndrome of infectious origin with organ failure |
| R652 | Systemic Inflammatory Response Syndrome of non-infectious origin without organ failure |
| R653 | Systemic Inflammatory Response Syndrome of non-infectious origin with organ failure |
| R659 | Systemic Inflammatory Response Syndrome, unspecified |

The ICD-10 codes correspond to the ones used by Buchman et al. (7).

eTable 2: monthly count and three-month rolling average of admissions between 2006 and 2018

|  |  | **2006** | **2007** | **2008** | **2009** | **2010** | **2011** | **2012** | **2013** | **2014** | **2015** | **2016** | **2017** | **2018** |
| --- | --- | --- | --- | --- | --- | --- | --- | --- | --- | --- | --- | --- | --- | --- |
| *monthly count* | | | |  |  |  |  |  |  |  |  |  |  |  |
|  | **Jan** | 98 | 162 | 128 | 145 | 149 | 179 | 222 | 252 | 261 | 249 | 313 | 349 | 947 |
|  | **Feb** | 124 | 129 | 130 | 133 | 147 | 189 | 198 | 251 | 230 | 243 | 292 | 362 | 815 |
|  | **Mar** | 124 | 168 | 140 | 153 | 171 | 193 | 206 | 250 | 261 | 281 | 326 | 536 | 735 |
|  | **Apr** | 128 | 136 | 154 | 157 | 155 | 195 | 188 | 233 | 258 | 278 | 314 | 786 | 591 |
|  | **May** | 124 | 155 | 166 | 170 | 186 | 198 | 244 | 253 | 276 | 290 | 315 | 820 | 657 |
|  | **Jun** | 129 | 143 | 139 | 164 | 185 | 209 | 193 | 248 | 272 | 273 | 290 | 826 | 606 |
|  | **Jul** | 137 | 135 | 150 | 176 | 195 | 214 | 247 | 280 | 304 | 289 | 296 | 861 | 690 |
|  | **Aug** | 144 | 163 | 158 | 148 | 155 | 209 | 233 | 266 | 276 | 298 | 314 | 898 | 728 |
|  | **Sep** | 148 | 157 | 134 | 123 | 164 | 216 | 228 | 255 | 274 | 325 | 302 | 919 | 658 |
|  | **Oct** | 123 | 149 | 166 | 171 | 182 | 197 | 244 | 289 | 304 | 287 | 350 | 948 | 752 |
|  | **Nov** | 142 | 138 | 164 | 194 | 206 | 228 | 232 | 263 | 272 | 295 | 326 | 876 | 621 |
|  | **Dec** | 127 | 140 | 167 | 179 | 197 | 221 | 234 | 287 | 318 | 333 | 353 | 985 | 643 |
|  | | | |  |  |  |  |  |  |  |  |  |  |  |
| *three month rolling average* | | | |  |  |  |  |  |  |  |  |  |  |  |
|  | **Jan** | 111 | 139 | 133 | 148 | 158 | 188 | 214 | 246 | 259 | 270 | 313 | 355 | 916 |
|  | **Feb** | 115 | 153 | 133 | 144 | 156 | 187 | 209 | 251 | 251 | 258 | 310 | 416 | 832 |
|  | **Mar** | 125 | 144 | 141 | 148 | 158 | 192 | 197 | 245 | 250 | 267 | 311 | 561 | 714 |
|  | **Apr** | 125 | 153 | 153 | 160 | 171 | 195 | 213 | 245 | 265 | 283 | 318 | 714 | 661 |
|  | **May** | 127 | 145 | 153 | 164 | 175 | 201 | 208 | 245 | 269 | 280 | 306 | 811 | 618 |
|  | **Jun** | 130 | 144 | 152 | 170 | 189 | 207 | 228 | 260 | 284 | 284 | 300 | 836 | 651 |
|  | **Jul** | 137 | 147 | 149 | 163 | 178 | 211 | 224 | 265 | 284 | 287 | 300 | 862 | 675 |
|  | **Aug** | 143 | 152 | 147 | 149 | 171 | 213 | 236 | 267 | 285 | 304 | 304 | 893 | 692 |
|  | **Sep** | 138 | 156 | 153 | 147 | 167 | 207 | 235 | 270 | 285 | 303 | 322 | 922 | 713 |
|  | **Oct** | 138 | 148 | 155 | 163 | 184 | 214 | 235 | 269 | 283 | 302 | 326 | 914 | 677 |
|  | **Nov** | 131 | 142 | 166 | 181 | 195 | 215 | 237 | 280 | 298 | 305 | 343 | 936 | 672 |
|  | **Dec** | 144 | 135 | 159 | 174 | 194 | 224 | 239 | 270 | 280 | 314 | 343 | 936 | 632 |

eTable 3. Inpatient hospital admissions with sepsis specific ICD-10 codes in Wales, all admissions and grouped by severity, 2006-2018

|  |  | **2006** | **2007** | **2008** | **2009** | **2010** | **2011** | **2012** | **2013** | **2014** | **2015** | **2016** | **2017** | **2018** |
| --- | --- | --- | --- | --- | --- | --- | --- | --- | --- | --- | --- | --- | --- | --- |
| **all admissions** | |  |  |  |  |  |  |  |  |  |  |  |  |  |
| total | | 1,548 | 1,775 | 1,796 | 1,913 | 2,092 | 2,448 | 2,669 | 3,127 | 3,306 | 3,441 | 3,791 | 9,166 | 8,443 |
| % change | | - | (14.7%) | (1.2%) | 6.5%) | (9.4%) | (17.0%) | (9.0%) | (17.2%) | (5.7%) | (4.1%) | (10.2%) | (141.8%) | (-7.9%) |
| **severe sepsis only** | |  |  |  |  |  |  |  |  |  |  |  |  |  |
| total | | 58 | 183 | 216 | 184 | 258 | 252 | 321 | 249 | 253 | 287 | 227 | 646 | 688 |
| % change | | - | (215.5%) | (18.0%) | (-14.8%) | (40.2%) | (-2.3%) | (27.4%) | (-22.4%) | (1.6%) | (13.4%) | (-20.9%) | (184.6%) | (6.5%) |

Admissions are presented by total numbers and percentage change compared to the previous year as baseline. Severe sepsis was categorized by admission to critical care.

eTable 4. Inpatient hospital admissions with sepsis specific ICD-10 codes in Wales, grouped by sex, age, areas of deprivation, frailty, comorbidity, length of stay and sepsis type, 2006-2018

|  |  | **2006** | **2007** | **2008** | **2009** | **2010** | **2011** | **2012** | **2013** | **2014** | **2015** | **2016** | **2017** | **2018** |
| --- | --- | --- | --- | --- | --- | --- | --- | --- | --- | --- | --- | --- | --- | --- |
| **sex** |  |  |  |  |  |  |  |  |  |  |  |  |  |  |
| female | total | 844 | 944 | 962 | 992 | 1108 | 1297 | 1339 | 1646 | 1690 | 1673 | 1923 | 4484 | 4059 |
|  | % change | - | (11.9%) | (1.9%) | (3.1%) | (11.7%) | (17.1%) | (3.2%) | (22.9%) | (2.7%) | (-1.0%) | (14.9%) | (133.2%) | (-9.5%) |
| male | total | 704 | 831 | 834 | 921 | 984 | 1151 | 1330 | 1481 | 1616 | 1768 | 1868 | 4682 | 4384 |
|  | % change | - | (18.0%) | (0.4%) | (10.4%) | (6.8%) | (17.0%) | (15.6%) | (11.4%) | (9.1%) | (9.4%) | (5.7%) | (150.6%) | (-6.4%) |
| **age** |  |  |  |  |  |  |  |  |  |  |  |  |  |  |
| under 65 | total | 444 | 540 | 513 | 543 | 680 | 779 | 836 | 973 | 1048 | 1090 | 1215 | 2523 | 2368 |
|  | % change | - | (21.6%) | (-5.0%) | (5.9%) | (25.2%) | (14.6%) | (7.3%) | (16.4%) | (7.7%) | (4.0%) | (11.5%) | (107.7%) | (-6.1%) |
| 65-74 | total | 294 | 339 | 361 | 400 | 447 | 491 | 578 | 738 | 825 | 891 | 911 | 2059 | 1971 |
|  | % change | - | (15.3%) | (6.5%) | (10.8%) | (11.8%) | (9.8%) | (17.7%) | (27.7%) | (11.8%) | (8.0%) | (2.2%) | (126.0%) | (-4.3%) |
| 75-84 | total | 453 | 501 | 501 | 544 | 534 | 643 | 692 | 803 | 833 | 833 | 983 | 2614 | 2394 |
|  | % change | - | (10.6%) | (0.0%) | (8.6%) | (-1.8%) | (20.4%) | (7.6%) | (16.0%) | (3.7%) | (0.0%) | (18.0%) | (165.9%) | (-8.4%) |
| 85+ | total | 357 | 395 | 421 | 426 | 431 | 535 | 563 | 613 | 600 | 627 | 682 | 1970 | 1710 |
|  | % change | - | (10.6%) | (6.6%) | (1.2%) | (1.2%) | (24.1%) | (5.2%) | (8.9%) | (-2.1%) | (4.5%) | (8.8%) | (188.9%) | (-13.2%) |
| **Deprivation fifth** |  |  |  |  |  |  |  |  |  |  |  |  |  |  |
| most deprived | total | 343 | 400 | 385 | 415 | 463 | 481 | 485 | 608 | 626 | 615 | 780 | 1883 | 1665 |
|  | % change | - | (16.6%) | (-3.8%) | (7.8%) | (11.6%) | (3.9%) | (0.8%) | (25.4%) | (3.0%) | (-1.8%) | (26.8%) | (141.4%) | (-11.6%) |
| 2 | total | 306 | 357 | 340 | 401 | 468 | 504 | 585 | 659 | 685 | 744 | 817 | 2021 | 1733 |
|  | % change | - | (16.7%) | (-4.8%) | (17.9%) | (16.7%) | (7.7%) | (16.1%) | (12.7%) | (4.0%) | (8.6%) | (9.8%) | (147.4%) | (-14.3%) |
| 3 | total | 334 | 383 | 392 | 395 | 409 | 538 | 614 | 664 | 721 | 777 | 810 | 1932 | 1836 |
|  | % change | - | (14.7%) | (2.4%) | (0.8%) | (3.5%) | (31.5%) | (14.1%) | (8.1%) | (8.6%) | (7.8%) | (4.3%) | (138.5%) | (-5.0%) |
| 4 | total | 336 | 341 | 342 | 337 | 386 | 485 | 523 | 663 | 680 | 707 | 762 | 1755 | 1760 |
|  | % change | - | (1.5%) | (0.3%) | (-1.5%) | (14.5%) | (25.7%) | (7.8%) | (26.8%) | (2.6%) | (4.0%) | (7.8%) | (130.3%) | (0.3%) |
| least deprived | total | 229 | 294 | 337 | 365 | 366 | 440 | 462 | 533 | 594 | 598 | 622 | 1575 | 1449 |
|  | % change | - | (28.4%) | (14.6%) | (8.3%) | (0.3%) | (20.2%) | (5.0%) | (15.4%) | (11.4%) | (0.7%) | (4.0%) | (153.2%) | (-8.0%) |
|  |  |  |  |  |  |  |  |  |  |  |  |  |  |  |
| **Frailty category** |  |  |  |  |  |  |  |  |  |  |  |  |  |  |
| Fit | total | 669 | 785 | 744 | 769 | 838 | 931 | 1069 | 1261 | 1365 | 1511 | 1632 | 3550 | 3472 |
|  | % change | - | (17.3%) | (-5.2%) | (3.4%) | (9.0%) | (11.1%) | (14.8%) | (18.0%) | (8.3%) | (10.7%) | (8.0%) | (117.5%) | (-2.2%) |
| Mild | total | 446 | 472 | 523 | 506 | 562 | 650 | 681 | 810 | 874 | 905 | 981 | 2610 | 2404 |
|  | % change | - | (5.8%) | (10.8%) | (-3.3%) | (11.1%) | (15.7%) | (4.8%) | (18.9%) | (7.9%) | (3.6%) | (8.4%) | (166.1%) | (-7.9%) |
| Moderate | total | 248 | 322 | 325 | 385 | 420 | 480 | 568 | 611 | 623 | 600 | 697 | 1896 | 1772 |
|  | % change | - | (29.8%) | (0.9%) | (18.5%) | (9.1%) | (14.3%) | (18.3%) | (7.6%) | (2.0%) | (-3.7%) | (16.2%) | (172.0%) | (-6.5%) |
| Severe | total | 0 | 0 | 130 | 169 | 186 | 272 | 247 | 308 | 312 | 283 | 331 | 897 | 725 |
|  | % change | - | (0.0%) | (0.0%) | (30.0%) | (10.1%) | (46.2%) | (-9.2%) | (24.7%) | (1.3%) | (-9.3%) | (17.0%) | (171.0%) | (-19.2%) |
| MISSING | total | 0 | 0 | 74 | 84 | 86 | 115 | 104 | 137 | 132 | 142 | 150 | 213 | 70 |
|  | % change | - | (0.0%) | (0.0%) | (13.5%) | (2.4%) | (33.7%) | (-9.6%) | (31.7%) | (-3.7%) | (7.6%) | (5.6%) | (42.0%) | (-67.1%) |
| **Comorbidity group** |  |  |  |  |  |  |  |  |  |  |  |  |  |  |
| LOW | total | 281 | 308 | 266 | 260 | 305 | 340 | 368 | 402 | 414 | 474 | 561 | 1419 | 1240 |
|  | % change | - | (9.6%) | (-13.6%) | (-2.3%) | (17.3%) | (11.5%) | (8.2%) | (9.2%) | (3.0%) | (14.5%) | (18.4%) | (152.9%) | (-12.6%) |
| MED | total | 333 | 392 | 381 | 397 | 464 | 519 | 576 | 658 | 738 | 769 | 753 | 1943 | 1926 |
|  | % change | - | (17.7%) | (-2.8%) | (4.2%) | (16.9%) | (11.9%) | (11.0%) | (14.2%) | (12.2%) | (4.2%) | (-2.1%) | (158.0%) | (-0.9%) |
| HIGH | total | 583 | 663 | 756 | 836 | 858 | 1137 | 1171 | 1365 | 1465 | 1440 | 1648 | 3890 | 3347 |
|  | % change | - | (13.7%) | (14.0%) | (10.6%) | (2.6%) | (32.5%) | (3.0%) | (16.6%) | (7.3%) | (-1.7%) | (14.4%) | (136.0%) | (-14.0%) |
| MISSING | total | 351 | 412 | 393 | 420 | 465 | 452 | 554 | 702 | 689 | 758 | 829 | 1914 | 1930 |
|  | % change | - | (17.4%) | (-4.6%) | (6.9%) | (10.7%) | (-2.8%) | (22.6%) | (26.7%) | (-1.9%) | (10.0%) | (9.4%) | (130.9%) | (0.8%) |
| **Length of stay** |  |  |  |  |  |  |  |  |  |  |  |  |  |  |
| 1-2 days | total | 249 | 299 | 287 | 317 | 343 | 407 | 405 | 480 | 541 | 581 | 605 | 1349 | 1261 |
|  | % change | - | (20.1%) | (-4.0%) | (10.5%) | (8.2%) | (18.7%) | (-0.5%) | (18.5%) | (12.7%) | (7.4%) | (4.1%) | (123.0%) | (-6.5%) |
| 3-4 days | total | 158 | 195 | 212 | 224 | 266 | 287 | 374 | 443 | 461 | 544 | 575 | 1304 | 1233 |
|  | % change | - | (23.4%) | (8.7%) | (5.7%) | (18.8%) | (7.9%) | (30.3%) | (18.5%) | (4.1%) | (18.0%) | (5.7%) | (126.8%) | (-5.4%) |
| 5-6 days | total | 152 | 199 | 220 | 243 | 228 | 315 | 360 | 439 | 457 | 466 | 529 | 1154 | 1090 |
|  | % change | - | (30.9%) | (10.6%) | (10.5%) | (-6.2%) | (38.2%) | (14.3%) | (21.9%) | (4.1%) | (2.0%) | (13.5%) | (118.2%) | (-5.6%) |
| 7+ days | total | 989 | 1082 | 1077 | 1129 | 1255 | 1439 | 1530 | 1765 | 1847 | 1850 | 2082 | 5359 | 4859 |
|  | % change | - | (9.4%) | (-0.5%) | (4.8%) | (11.2%) | (14.7%) | (6.3%) | (15.4%) | (4.7%) | (0.2%) | (12.5%) | (157.4%) | (-9.3%) |
| **Sepsis type** |  |  |  |  |  |  |  |  |  |  |  |  |  |  |
| organism specific | total | 671 | 857 | 720 | 705 | 791 | 828 | 823 | 974 | 926 | 851 | 888 | 2235 | 2122 |
|  | % change | - | (27.7%) | (-16.0%) | (-2.1%) | (12.2%) | (4.7%) | (-0.6%) | (18.4%) | (-4.9%) | (-8.1%) | (4.4%) | (151.7%) | (-5.1%) |
| unspecified | total | 877 | 918 | 1076 | 1208 | 1301 | 1620 | 1846 | 2153 | 2380 | 2590 | 2903 | 6931 | 6321 |
|  | % change | - | (4.7%) | (17.2%) | (12.3%) | (7.7%) | (24.5%) | (14.0%) | (16.6%) | (10.5%) | (8.8%) | (12.1%) | (138.8%) | (-8.8%) |
|  |  |  |  |  |  |  |  |  |  |  |  |  |  |  |

Admissions are presented by total numbers and percentage change compared to the previous year as baseline. Areas of deprivation are categorised by the Welsh Index of Multiple Deprivation, 2014; from 1 - most deprived to 5 – least deprived, respectively (7). The frailty status was based on the eFI. It assigns a frailty score to an individual calculated using 36 variables from primary care data, including symptoms, signs, diseases, disabilities and abnormal laboratory values Scores were calculated for individuals on their admission date and categorised as: fit (0 - 0.12), mild (>0.12 – 0.24), moderate (>0.24 – 0.36), and severely frail (>0.36), respectively (5).

Comorbidity was grouped by the Charlson Comorbidity Index (6). Scores were categorised into three groups, by number of comorbidities present: low (-1-0), medium (1-10) and high (>10), respectively.

Sepsis type was grouped by specific organisms coded episodes versus unspecified episodes.

eTable 5. Inpatient bed days and associated costs of sepsis admissions

| Admission Year | Number of inpatient days | Patients admitted (n) | Number of admissions | Cost (£) |
| --- | --- | --- | --- | --- |
| 2006 | 28,151 | 1,509 | 1,599 | 13,259,121 |
| 2007 | 31,801 | 1,680 | 1,824 | 14,978,271 |
| 2008 | 31,277 | 1,712 | 1,850 | 14,731,467 |
| 2009 | 32,383 | 1,811 | 1,964 | 15,252,393 |
| 2010 | 34,511 | 1,950 | 2,155 | 16,254,681 |
| 2011 | 39,941 | 2,268 | 2,506 | 18,812,211 |
| 2012 | 42,271 | 2,490 | 2,734 | 19,909,641 |
| 2013 | 47,583 | 2,889 | 3,209 | 22,411,593 |
| 2014 | 48,917 | 3,023 | 3,392 | 23,039,907 |
| 2015 | 48,882 | 3,164 | 3,550 | 23,023,422 |
| 2016 | 56,614 | 3,482 | 3,914 | 26,665,194 |
| 2017 | 152,560 | 8,172 | 9,410 | 71,855,760 |
| 2018 | 127,701 | 7,626 | 8,708 | 60,147,171 |

Inpatient care treatment cost estimations were calculated from the National Schedule of reference costs 2017/18 (8).

eTable 6: number and percent of admissions by sepsis diagnosis category

| Severity Levels | Description | ICD-10 codes | number of admissions | percent of admissions |
| --- | --- | --- | --- | --- |
|  |  |  |  |  |
| non-severe sepsis, organism specific | Salmonella sepsis | A021 | 29 | 0.1% |
|  | Septicaemic plague | A207 | - | - |
|  | Anthrax sepsis | A227 | - | - |
|  | Erysipelothrix sepsis | A267 | * |  |
|  | Listerial sepsis | A327 | 24 | 0.1% |
|  | Sepsis due to streptococcus, group A | A400 | 185 | 0.5% |
|  | Sepsis due to streptococcus, group B | A401 | 144 | 0.4% |
|  | Sepsis due to streptococcus, group D | A402 | 55 | 0.2% |
|  | Sepsis due to Streptococcus pneumoniae | A403 | 260 | 0.7% |
|  | Other streptococcal sepsis | A408 | 456 | 1.2% |
|  | Streptococcal sepsis, unspecified | A409 | 240 | 0.7% |
|  | Sepsis due to Staphylococcus aureus | A410 | 1637 | 4.5% |
|  | Sepsis due to other specified staphylococcus | A411 | 760 | 2.1% |
|  | Sepsis due to unspecified staphylococcus | A412 | 200 | 0.5% |
|  | Sepsis due to Haemophilus influenzae | A413 | 38 | 0.1% |
|  | Sepsis due to anaerobes | A414 | 91 | 0.2% |
|  | Sepsis due to other Gram-negative organisms | A415 | 4489 | 12.3% |
|  | Other specified sepsis | A418 | 1609 | 4.4% |
|  | Actinomycotic sepsis | A427 | - | - |
|  | Other gonococcal infections | A5486 | - | - |
|  | Candidal sepsis | B377 | 32 | 0.1% |
|  | Puerperal sepsis | O85X | 771 | 2.1% |
|  |  |  |  |  |
|  |  |  |  |  |
| non-severe sepsis, unspecified | Sepsis, unspecified | A419 | 25585 | 69.9% |
|  | Systemic Inflammatory Response Syndrome of infectious origin with organ failure | R651 | * |  |
|  |  |  |  |  |
|  |  |  |  |  |
| severe sepsis, without shock | Systemic Inflammatory Response Syndrome of infectious origin without organ failure | R6520 | - | - |
|  |  |  |  |  |
|  |  |  |  |  |
| septic shock | Systemic Inflammatory Response Syndrome of infectious origin with organ failure | R6521 | - | - |

eTable 7. The ten most common coded main causes of death for the sepsis cases

| ICD-10 code | Main cause for death | Cases |
| --- | --- | --- |
| A419 | Sepsis, unspecified | 4936 |
| J189 | Pneumonia, unspecified | 2077 |
| J180 | Bronchopneumonia, unspecified | 1869 |
| R688 | Other specified general symptoms and signs | 784 |
| C349 | Malignant neoplasm: Bronchus or lung, unspecified | 614 |
| N390 | Urinary tract infection, site not specified | 576 |
| J690 | Pneumonitis due to food and vomit | 497 |
| C798 | Secondary malignant neoplasm of other specified sites | 409 |
| C80 | Malignant neoplasm without specification of site | 381 |
| I219 | Acute myocardial infarction, unspecified | 350 |

eFigure 1. Inpatient hospital admissions with sepsis specific ICD-10 codes in Wales, grouped by age, 2006-2018


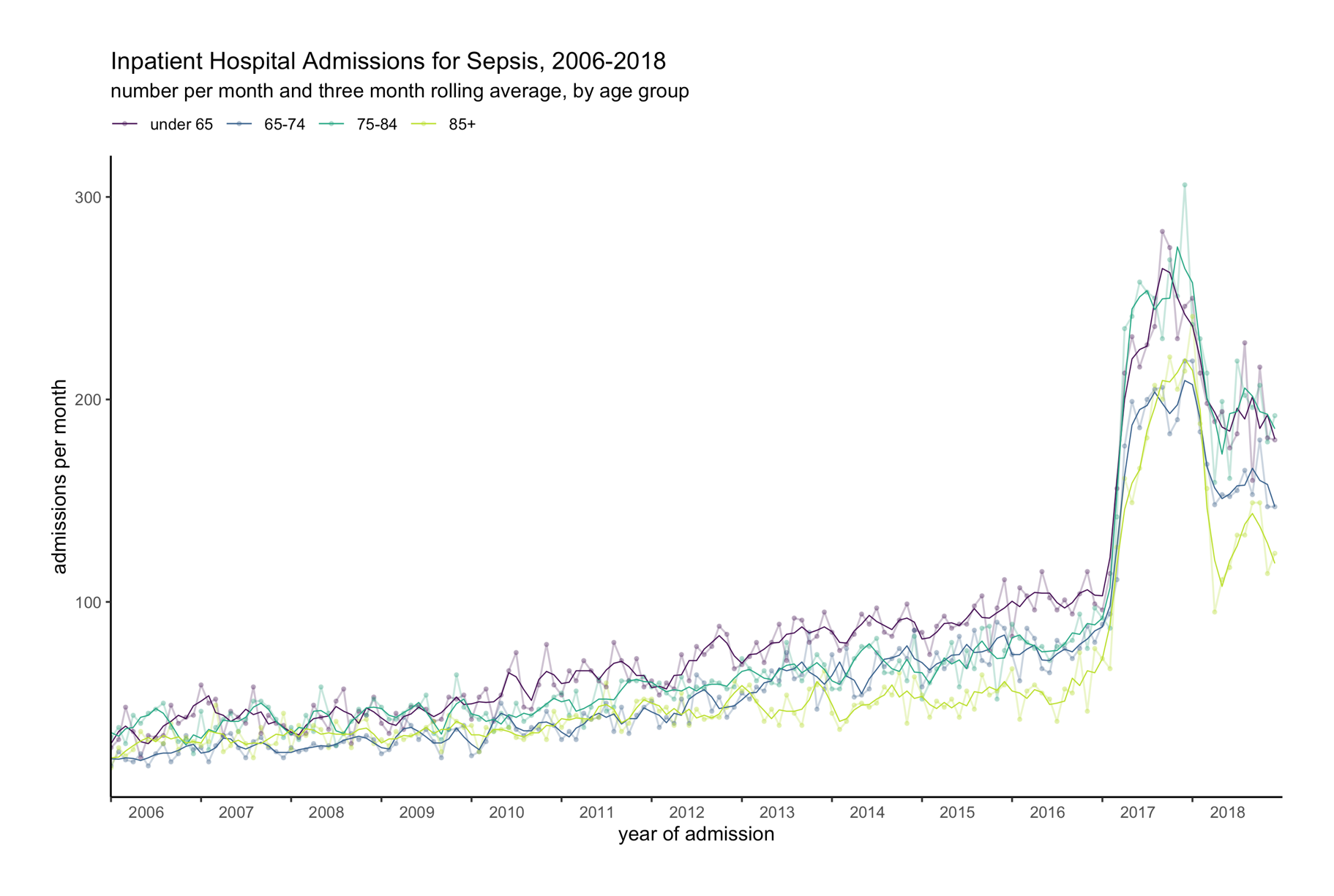


eFigure 2. Inpatient hospital admissions with sepsis specific ICD-10 codes in Wales, grouped by sex, 2006-2018


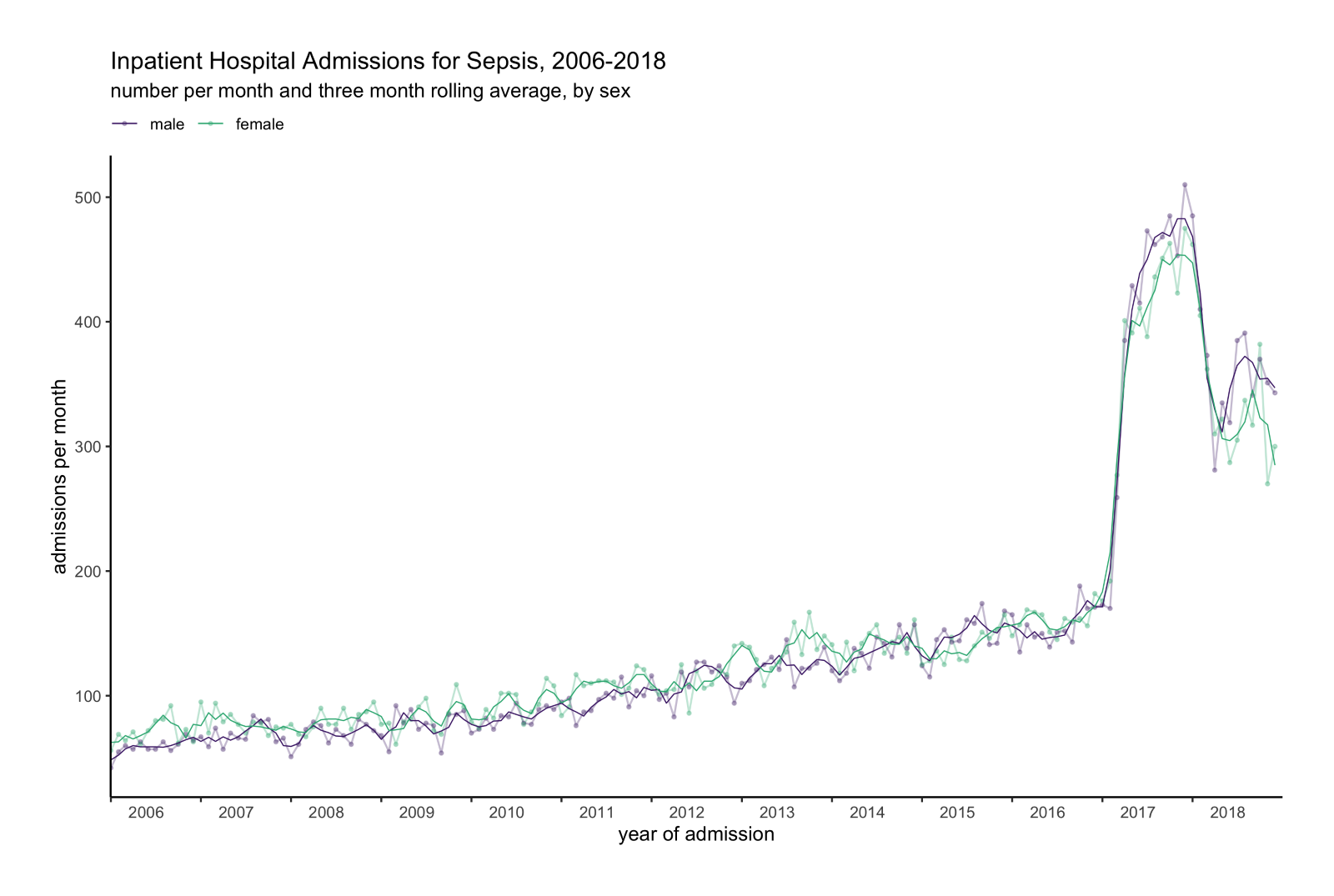


eFigure 3. Inpatient hospital admissions with sepsis specific ICD-10 codes in Wales, grouped by areas of deprivation, 2006-2018


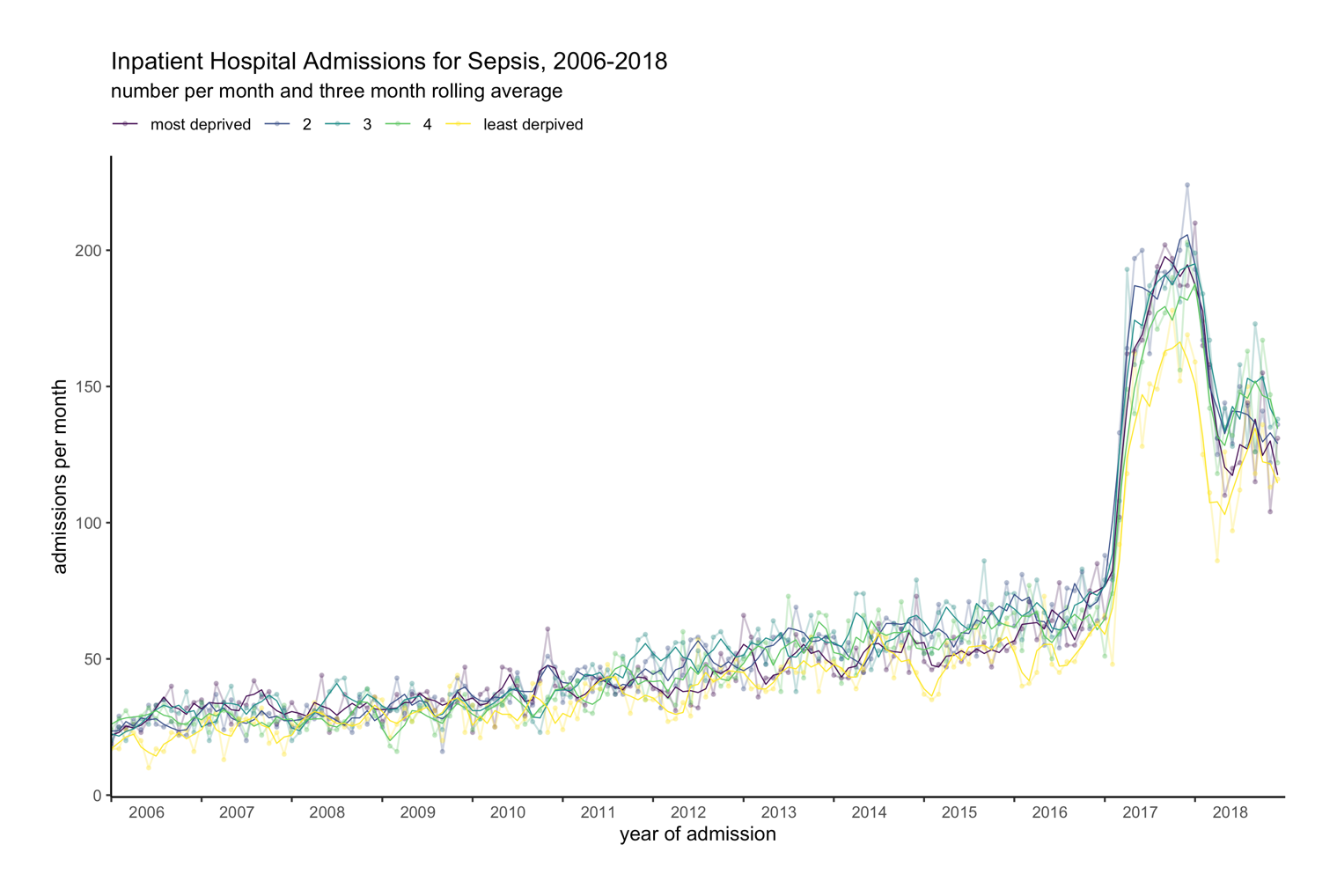


Areas of deprivation are categorised by the Welsh Index of Multiple Deprivation, 2014; from 1 - most deprived to 5 – least deprived, respectively (7).

eFigure 4. Inpatient hospital admissions with sepsis specific ICD-10 codes in Wales, grouped by levels of comorbidity, 2006-2018


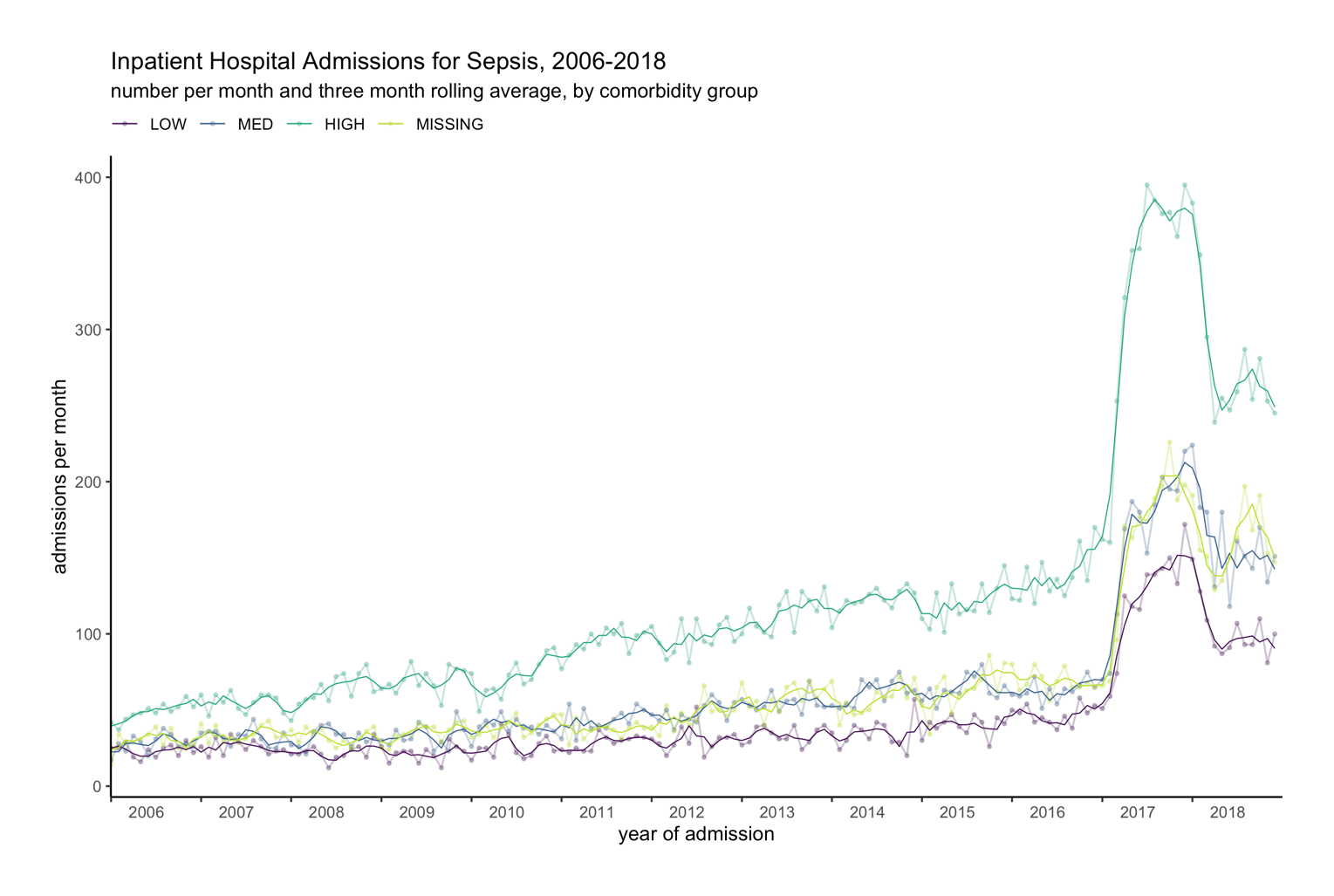


Comorbidity was grouped by the Charlson Comorbidity Index (6). Scores were categorised into three groups, by number of comorbidities present: low (-1-0), medium (1-10) and high (>10), respectively.

eFigure 5. Inpatient hospital admissions with sepsis specific ICD-10 codes in Wales, grouped by presence or absence of specific causative organism, 2006-2018


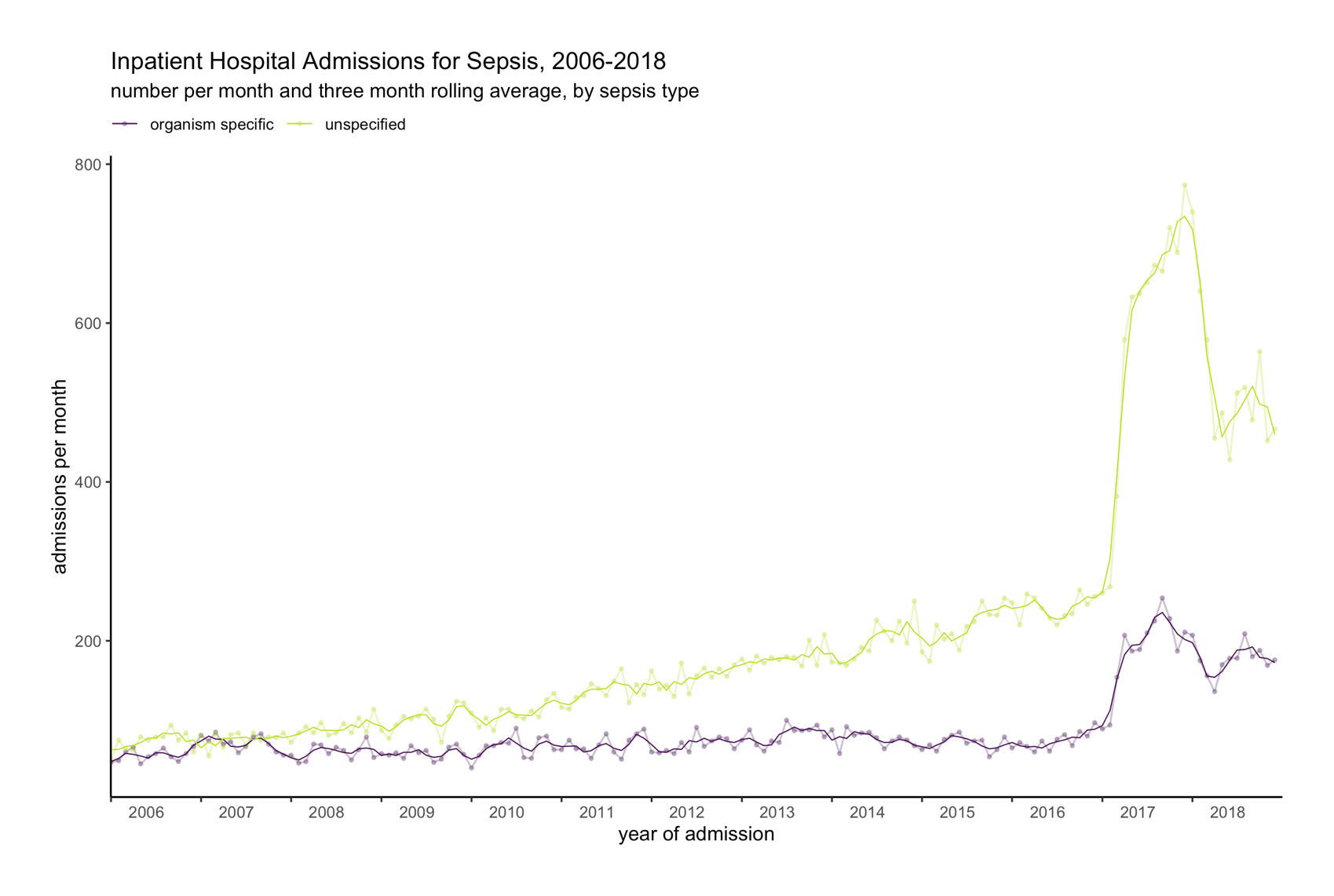


The breakdown of ICD-10 codes for the causative organisms is provided in eTable 5.

eFigure 6. Inpatient hospital admissions with sepsis specific ICD-10 codes in Wales, grouped by frailty status, 2006-2018


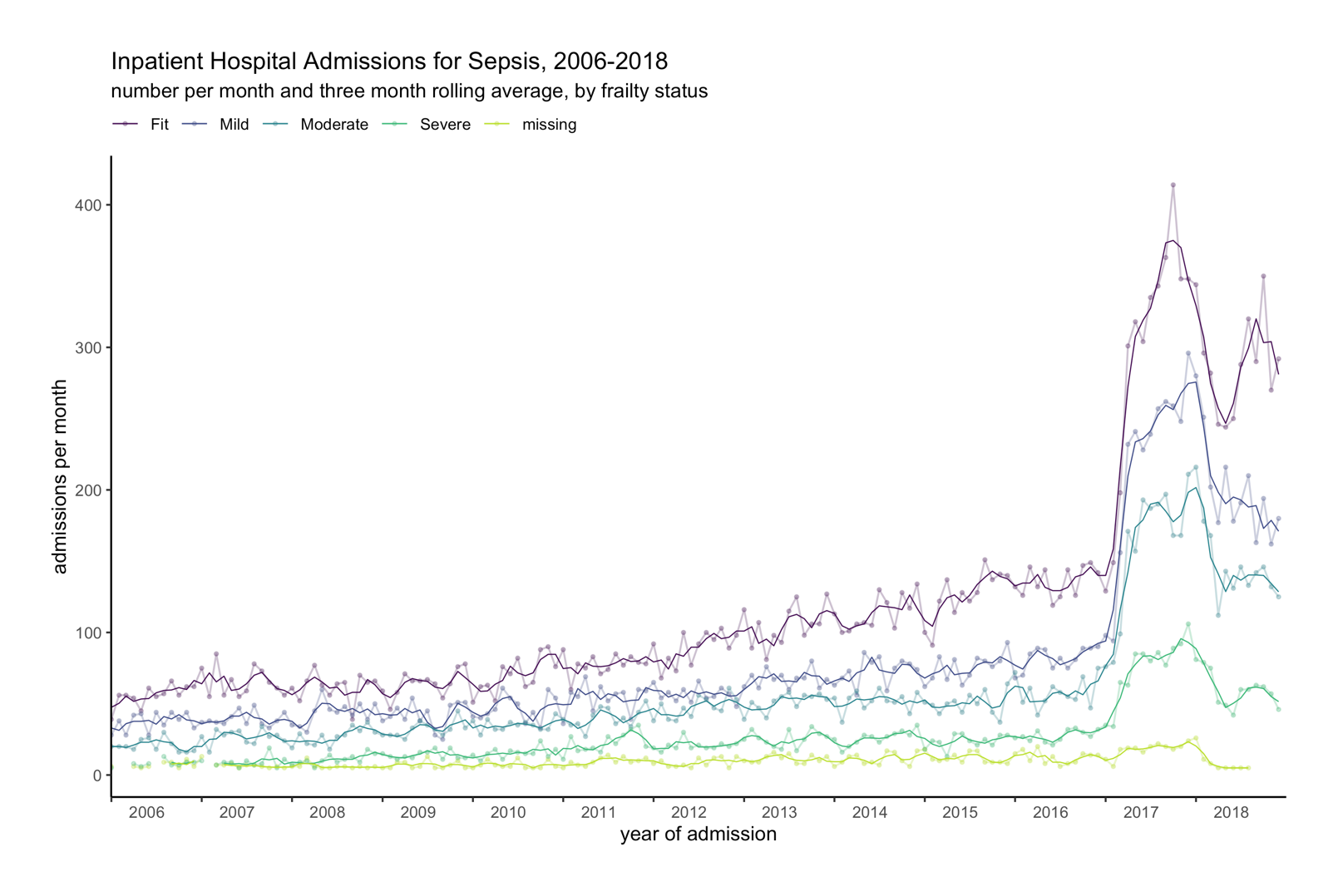


The frailty status was based on the eFI. It assigns a frailty score to an individual calculated using 36 variables from primary care data, including symptoms, signs, diseases, disabilities and abnormal laboratory values Scores were calculated for individuals on their admission date and categorised as: fit (0 - 0.12), mild (>0.12 – 0.24), moderate (>0.24 – 0.36), and severely frail (>0.36), respectively (5).

eFigure 7. Inpatient hospital admissions with sepsis specific ICD-10 codes in Wales, grouped by length of stay, 2006-2018


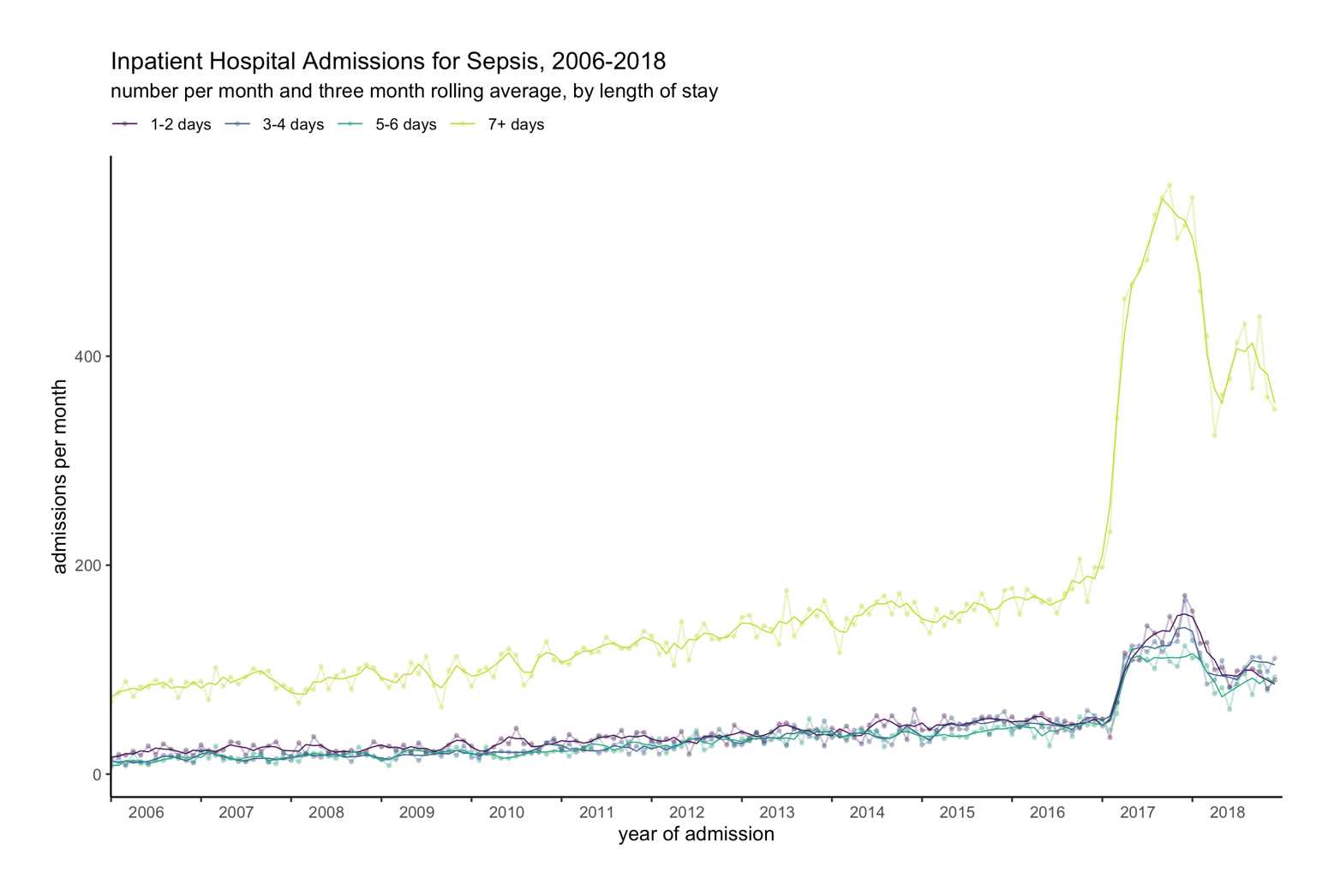


eFigure 8. Mortality rates of patients admitted with sepsis specific ICD-10 codes in Wales, grouped by age, 2006-2018
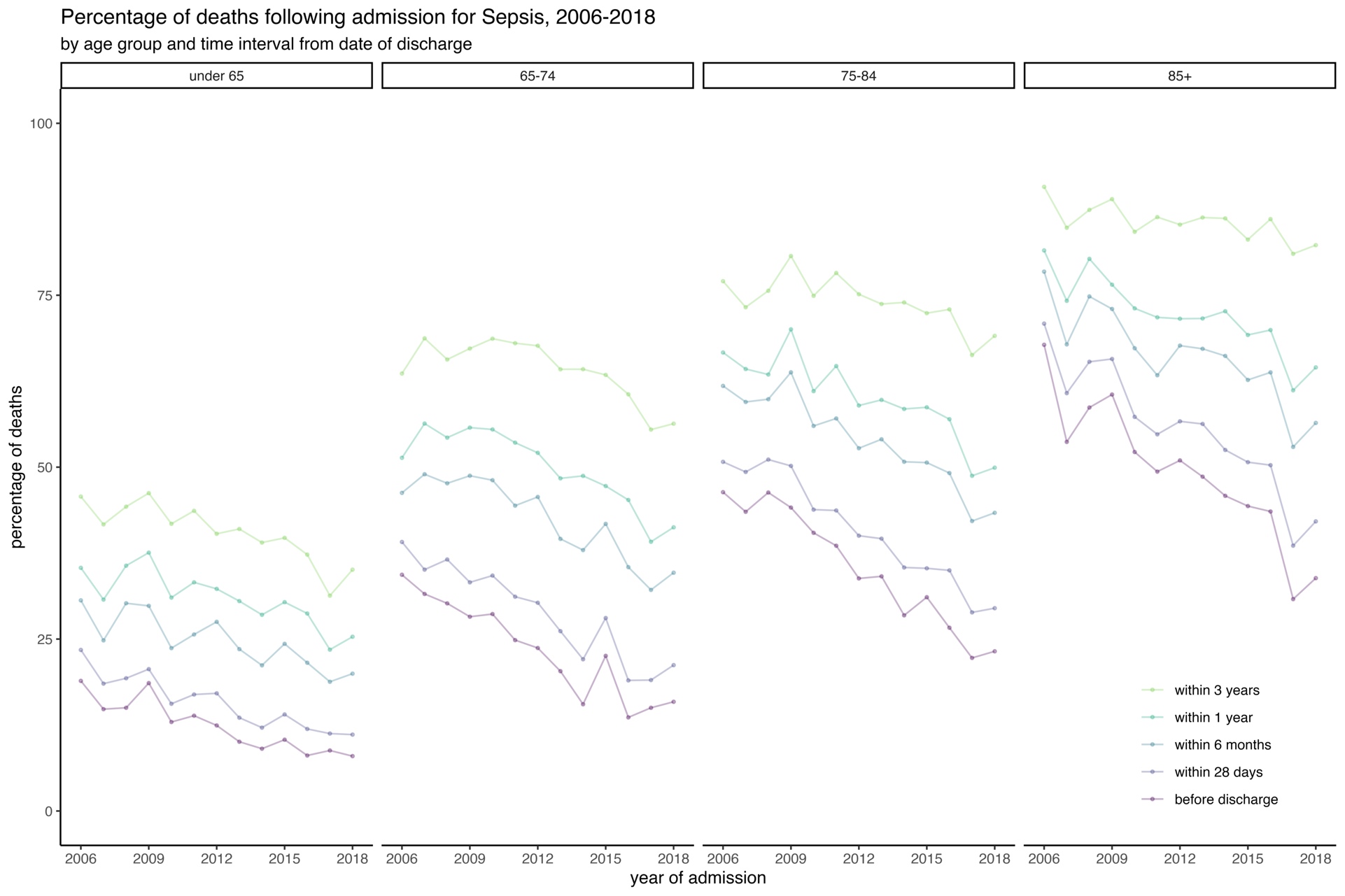


References

1. Lyons RA, Jones KH, John G, et al.: The SAIL databank: linking multiple health and social care datasets. *BMC Med Inform Decis Mak* 2009; 9:3

2. Jones KH, Ford DV, Jones C, et al.: A case study of the Secure Anonymous Information Linkage (SAIL) Gateway: a privacy-protecting remote access system for health-related research and evaluation. *J Biomed Inform* 2014; 50:196–204

3. Ford DV, Jones KH, Verplancke J-P, et al.: The SAIL Databank: building a national architecture for e-health research and evaluation. *BMC Health Serv Res* 2009; 9:157

4. <https://statswales.gov.wales/Catalogue/Community-Safety-and-Social-Inclusion/Welsh-Index-of-Multiple-Deprivation/Archive/WIMD-2014> accessed on accessed on November 7^th^ 2023

5. Clegg A, Bates C, Young J, et al.: Development and validation of an electronic frailty index using routine primary care electronic health record data. *Age Ageing* 2016; 45:353–360

6. Bottle A, Aylin P: Comorbidity scores for administrative data benefited from adaptation to local coding and diagnostic practices. *J Clin Epidemiol* 2011; 64:1426–1433

7. Buchman TG, Simpson SQ, Sciarretta KL, et al.: Sepsis Among Medicare Beneficiaries: 3. The Methods, Models, and Forecasts of Sepsis, 2012-2018. *Crit Care Med* 2020; 48:302–318

8. Archived Reference Costs | NHS Improvement [Internet]. [accessed on November 7^th^ 2023] Available from: https://webarchive.nationalarchives.gov.uk/ukgwa/20200501111106/https://improvement.nhs.uk/resources/reference-costs/
